# *Saccharomyces cerevisiae* strains performing similarly during fermentation of lignocellulosic hydrolysates show pronounced differences in transcriptional stress responses

**DOI:** 10.1128/aem.02330-23

**Published:** 2024-04-08

**Authors:** Elena Cámara, Maurizio Mormino, Verena Siewers, Yvonne Nygård

**Affiliations:** 1 Division of Industrial Biotechnology, Department of Life Sciences, Chalmers University of Technology, Gothenburg, Sweden; 2 Division of Systems and Synthetic Biology, Department of Life Sciences, Chalmers University of Technology, Gothenburg, Sweden; 3 VTT Technical Research Centre of Finland, Espoo, Finland; Danmarks Tekniske Universitet, The Novo Nordisk Foundation Center for Biosustainability, Kgs. Lyngby, Denmark

**Keywords:** industrial yeast strains, wild-type isolates, RNA sequencing, inhibitor stress, tolerance

## Abstract

Improving our understanding of the transcriptional changes of *Saccharomyces cerevisiae* during fermentation of lignocellulosic hydrolysates is crucial for the creation of more efficient strains to be used in biorefineries. We performed RNA sequencing of a CEN.PK laboratory strain, two industrial strains (KE6-12 and Ethanol Red), and two wild-type isolates of the LBCM collection when cultivated anaerobically in wheat straw hydrolysate. Many of the differently expressed genes identified among the strains have previously been reported to be important for tolerance to lignocellulosic hydrolysates or inhibitors therein. Our study demonstrates that stress responses typically identified during aerobic conditions such as glutathione metabolism, osmotolerance, and detoxification processes also are important for anaerobic processes. Overall, the transcriptomic responses were largely strain dependent, and we focused our study on similarities and differences in the transcriptomes of the LBCM strains. The expression of sugar transporter-encoding genes was higher in LBCM31 compared with LBCM109 that showed high expression of genes involved in iron metabolism and genes promoting the accumulation of sphingolipids, phospholipids, and ergosterol. These results highlight different evolutionary adaptations enabling *S. cerevisiae* to strive in lignocellulosic hydrolysates and suggest novel gene targets for improving fermentation performance and robustness.

**IMPORTANCE:**

The need for sustainable alternatives to oil-based production of biochemicals and biofuels is undisputable. *Saccharomyces cerevisiae* is the most commonly used industrial fermentation workhorse. The fermentation of lignocellulosic hydrolysates, second-generation biomass unsuited for food and feed, is still hampered by lowered productivities as the raw material is inhibitory for the cells. In order to map the genetic responses of different *S. cerevisiae* strains, we performed RNA sequencing of a CEN.PK laboratory strain, two industrial strains (KE6-12 and Ethanol Red), and two wild-type isolates of the LBCM collection when cultivated anaerobically in wheat straw hydrolysate. While the response to inhibitors of *S. cerevisiae* has been studied earlier, this has in previous studies been done in aerobic conditions. The transcriptomic analysis highlights different evolutionary adaptations among the different *S. cerevisiae strains* and suggests novel gene targets for improving fermentation performance and robustness.

## INTRODUCTION

The production of renewable chemicals and fuels from lignocellulosic hydrolysates, made from biomass unsuited for food and feed, is an important part of a forward-looking climate policy where fossil raw materials are replaced with biological resources. The treatments required to free the monomeric sugars from the lignocellulosic biomass however leads to the release of several compounds that are inhibitory for microorganisms used as biotechnological production hosts. When *Saccharomyces cerevisiae* is cultivated in lignocellulosic hydrolysates, its growth and ethanol production are challenged by high concentrations of different inhibitors such as furfural, weak acids, and phenolics. In spite of the vast number of studies and genetic modifications performed on *S. cerevisiae* to improve its tolerance [reviewed in reference (1)], overcoming the inhibitor stress still remains a challenge for second-generation biorefineries that convert lignocellulosic biomass into biochemicals.

In the past decades, considerable amounts of resources have been invested into isolation of new yeast strains with higher tolerance towards lignocellulosic hydrolysate inhibitors. Wild yeasts collected from harsh habitats may have developed superior stress tolerance, due to the selective pressures of their environment. Therefore, those wild yeasts may represent excellent starting points to develop inhibitor-tolerant cell factories (2). The manipulation and genetic improvement of such strains may however be more challenging compared with those of the laboratory strains that are commonly used also in studies aiming at improving industrially relevant stress responses (1). This is partly due to less knowledge of their physiology and genetics but also due to industrial strains often being diploids, tetraploids, and even euploids. Strains used in industrial settings display typical phenotypic traits such as high ethanol yield, thermostability, and increased inhibitor tolerance, which make them suited for large-scale bioprocesses (3, 4). Notably, many strains with higher tolerance have been developed through classical strain engineering such as adaptive laboratory evolution. This means that the genetics behind a tolerant phenotype may not be evident.

Numerous studies on the genetic responses of yeast subjected to stress caused by lignocellulosic hydrolysates have already been conducted. There are several studies investigating the transcriptomic response of *S. cerevisiae* subjected to a single inhibitor, including furfural (5–7), acetic acid (5, 8, 9), formic acid (10, 11), and hydroxymethylfurfural (HMF) (9, 12, 13). Previous studies have also addressed the transcriptomic responses to mixtures of inhibitors (14, 15). Moreover, transcriptomic studies of cells grown in the presence of hardwood spent sulfite liquor (15) or of cells during propagation in lignocellulosic hydrolysates (16) have been conducted. While the tolerance and response to individual inhibitors differ, it is also known that the inhibitors may have synergistic effects (17). Moreover, complex media such as lignocellulosic hydrolysates contain not only the main inhibitors but often also lesser amounts of other compounds that may be harmful for the cells. The cumulative effect of all the compounds found in a specific lignocellulosic hydrolysate may thus not be observed in cells grown in the presence of synthetic inhibitor mixtures. A further complicating matter is strain-dependent variance in tolerance and response to lignocellulosic hydrolysates (1). Remarkably, while many biotechnological production processes including bioethanol production are run anaerobically, the transcriptomic studies on hydrolysates have so far been conducted in aerobic conditions.

In this study, we investigate the transcriptomes of five *S*. *cerevisiae* strains, one laboratory strain, two industrial strains, and two wild-type isolates. RNA sequencing was conducted for strains grown in wheat straw hydrolysate (WSH) under anaerobic conditions. Differences as well as common traits emerged from the transcriptome comparisons. In particular, we aimed to determine what transcriptional response enabled the good performance of the LBCM strains that performed as well as the industrially adapted strains. The results presented aid in understanding the mechanisms behind lignocellulosic hydrolysate tolerance in yeast and provide new intel to engineer novel strains suitable for biorefinery applications.

## MATERIALS AND METHODS

### Strains, media, and culture conditions

Five *S*. *cerevisiae* strains were used in this study, a commonly used laboratory strain of the CEN.PK linage, two industrial strains used for bioethanol production, and two strains isolated from cachaça distilleries (Table 1). The industrial strain KE6-12 is derived from TMB400 (Albers et al., unpublished).

**TABLE 1:** *S. cerevisiae* strains used in this study

Strain	Description	Reference or source
CEN.PK113-7D	Haploid laboratory strain	(18)
Ethanol Red	Diploid industrial strain; commercially used for bioethanol production	Fermentis, USA
KE6-12	Diploid industrial strain expressing X*YL1* and *XYL2* from *Pichia stipitis* and overexpressing the endogenous *XKS1*. The strain is derived from TMB400 and has been subjected to evolutionary engineering for improved xylose fermentation efficiency and lignocellulosic inhibitor tolerance.	Albers et al., unpublished
LBCM31	Strain isolated from a cachaça distillery	(19)
LBCM109	Strain isolated from a cachaça distillery	(19)

The strains were maintained in yeast extract peptone dextrose (YPD) medium containing 10 g L^−1^ yeast extract, 20 g L^−1^ peptone, and 20 g L^−1^ glucose. The strains were grown in shake flasks in liquid minimal medium containing 70% (wt/wt) of WSH and 3 g L^−1^ potassium phosphate, 2.4 g L^−1^ urea, 0.5 g L^−1^ magnesium phosphate, 10.2 g L^−1^ k-phthalate, 1 mL L^−1^ trace metal solution, and 1 mL L^−1^ vitamin solution (20). The medium was sterilized using 0.2 µm nylon membrane filters, and the pH was adjusted to 5.5 with 5 M NaOH. The WSH was prepared as described by van Dijk et al. (21) and contained 80.3 g/L glucose, 31.7 g L^−1^ xylose, 4.7 g L^−1^ arabinose, 8.4 g L^−1^ acetic acid, 0.6 g L^−1^ HMF, and 4.6 g L^−1^ furfural.

Precultures were inoculated from glycerol cryostocks and incubated overnight at 30°C and 200 rpm in YPD. After reaching a stationary phase, 100-mL shake flasks were inoculated at an initial optical density at 600 nm (OD_600_) of 1, with 9.3 mL of WSH medium at 75% and 0.7 mL of preculture. In YPD and aerobic conditions, all strains grew similarly. Pre-cultivation in WSH was not done, as the growth among the strains grown aerobically in WSH was very varying (data not shown). For anaerobic cultures, non-baffled shake flasks were chosen, and the medium was gassed with N_2_ for 10 s after inoculation. An air trap filled with sterile glycerol was used to prevent oxygen diffusion. Cultures were incubated at 30°C and 200 rpm and the growth was monitored online with a Cell Growth Quantifier (Aquila biolabs, Germany). A standard curve to correlate backscatter and OD_600_ was prepared following the instructions of the manufacturer. Quadruplicate cultures were carried out for each strain, and samples were taken within 2 h after the culture had reached stationary phase.

### Determination of maximal growth rate, OD_600_, and dry cell weight

The maximal specific growth rate (µmax) for each strain was defined according to the following equation:


$$\mu max=\frac{ln\left(x2/x1\right)}{t2-t1}$$

where *x*2 and *x*1 are the manually identified finishing and starting OD_600_ values of the growth curve at its highest slope, respectively, and *t*2 and *t*1 the corresponding time points. The OD_600_ value was determined from cells resuspended in deionized water. The cells were harvested from 1.5 mL of culture by centrifugation. The OD_600_ was determined in triplicate for each sample by measuring the absorbance at 600 nm using a Genesys 20 spectrophotometer (Thermo Scientific, USA). The biomass concentration was also determined as dry cell weight (DCW) by collecting cells from 1.5 mL of culture by centrifugation, followed by resuspension in 1.5 mL of deionized water, and filtration using pre-weighed 0.45-µm polyether sulfone membranes (Sartorius, Germany). The filters were dried for 2 h at 65°C and weighed after 2 days in a desiccator.

### Extracellular metabolite quantification

Culture samples were filtered through 0.2-µm nylon membrane filters (VWR, USA) and supernatants were used for extracellular metabolite quantification by high-performance liquid chromatography, using a refractive index detector (Jasco, Italy). Glucose, xylose, arabinose, acetic acid, HMF, and furfural were separated using a Rezex ROA-Organic Acid H^+^ column (Phenomenex, Germany) at a flow rate of 0.8 mL min^−1^, at 80°C, using 5 mM sulfuric acid as eluent.

### RNA extraction and quality control

Samples for RNA extraction were harvested by centrifugation, and pellets were frozen in liquid nitrogen and stored at −80°C until extraction as previously described (22). The concentration and quality of the RNA were assessed using the NanoDrop 2000 Spectrophotometer (Thermo Scientific, USA), and the integrity was confirmed using the 2100 Bioanalyzer System with the RNA 6000 Nano Assay (Agilent Technologies, USA). Four replicate samples with an RNA integrity number above eight were used for the library preparation.

### Library preparation and RNA sequencing

Library preparation and sequencing were performed at the SNP&SEQ Technology Platform (Uppsala, Sweden). Sequencing libraries were prepared from 500 ng total RNA using the TruSeq Stranded mRNA Library Preparation Kit (cat# 20020595, Illumina Inc., USA), including polyA selection, following the instructions of the manufacturer (protocol #1000000040498). Unique dual indexes (cat# 20022371, Illumina Inc., USA) were used. The libraries were sequenced using a NovaSeq 6000 system (Illumina Inc., USA) and a SP-200 flow cell with pair-end 100 bp read length and v1.5 sequencing chemistry. A sequencing library for the phage PhiX was included as a 1% spike-in in the sequencing run. The sequencing generated a coverage of 8 to 14 M reads per library.

### Pre-analysis and data quality control

Raw data from the RNA sequencing were analyzed using the nf-core rnaseq pipeline release 1.4.2 (23). Briefly, the workflow processed the raw data from FastQ inputs, aligned the reads, generated counts relative to genes or transcripts, and performed an extensive quality control of the results. Quality score distribution across the reads was assessed with FastQC v0.11.8 (24) followed by the removal of adapter contamination and trimming of low-quality regions with TrimGalore v0.6.4 (25, 26). The RSeQC v3.0.1 package (27) was used to evaluate the parameters read distribution, inner distance, read duplication, junction saturation, and infer experiment. Duplication rates for genes were analyzed using dupRadar v1.14.0 (28), and the complexity of the libraries was estimated using Preseq v2.0.3 (29). Reads were mapped to the reference genome R64-1-1 using vSTAR_2.6.1d (30), while the featureCounts v1.6.4 package (31) was selected to obtain counts of reads mapping to genes. The quality control output files were visualized using MultiQC v1.7 (32).

### Differential gene expression and functional enrichment analysis

Gene counts were imported into R, and all subsequent analyses of differential gene expression (DGE) were done using the EdgeR package (33). Exploratory analysis to investigate sample similarities was performed through multi-dimensional scaling plots using the *plotMDS* function. Genes with low expression values were filtered out using the *filterbyExpr* function, followed by a normalization with the weighted trimmed mean of M-values using the *calcNormFactor* function. Gene dispersion was calculated using pairwise contrasts with the *estimateDisp* function. To evaluate the differentially expressed genes (DEGs), the function *makeContrasts* was selected, controlling the false discovery rate (FDR) using a Benjamini-Hochberg correction (FDR < 0.01).

Gene Ontology (GO) enrichment analysis was performed using the R package PIANO (Platform for Integrative Analysis of Omics) (34), using gene level statistics. Only genes that passed the threshold of an adjusted *P* value < 0.01 were selected for the analysis, and the limits of genes per cluster were set to 5 and 500. The code used for the analysis is available at GitHub (https://github.com/MorMauri/Transcriptomics-WSH). Pathway genes are presented according to the KEGG Pathway database (https://www.genome.jp/kegg/pathway.html).

## RESULTS AND DISCUSSION

### All strains grew similarly in wheat straw hydrolysate

**Fig 1:**
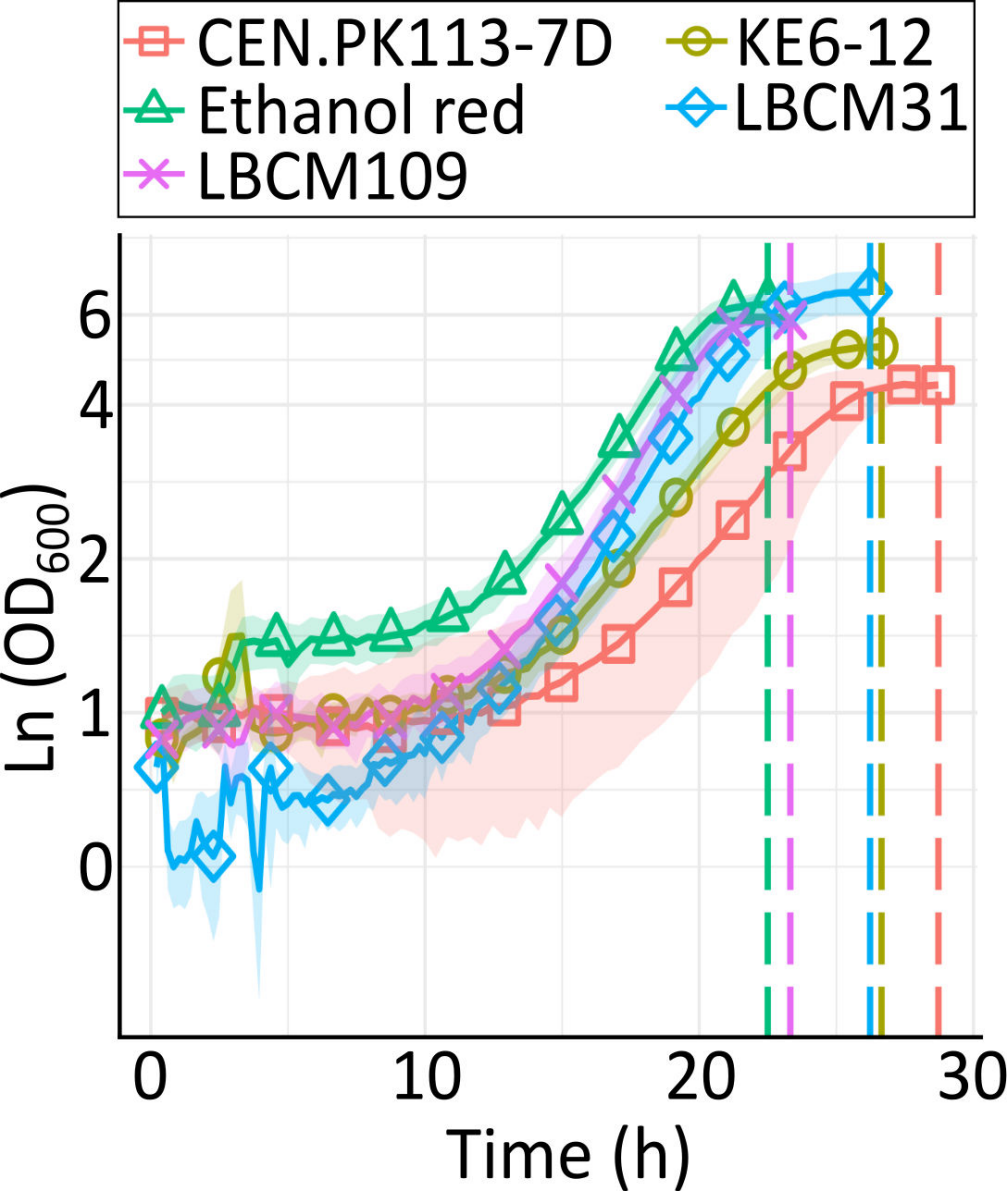
Anaerobic cultivation of CEN.PK113-7D (red squares), KE6-12 (yellow circles), Ethanol Red (green triangles), LBCM31 (blue diamonds), and LBCM109 (purple crosses) in minimal medium containing 70% WSH. Sampling time for each culture is indicated by the vertical dashed line in the corresponding color. Data obtained from four biological replicates; shadows show the standard deviation.

In order to investigate strain-dependent transcriptional responses to growth in WSH, five *S*. *cerevisiae* strains (Table 1) were grown in minimal medium supplemented with 70% WSH. The strains included the laboratory strain CEN.PK113-7D, the industrial strains Ethanol Red (Fermentis, USA) and KE6-12 (Albers et al., unpublished), and two wild-type strains (19). The strains were grown anaerobically in batch cultures and sampled for RNA sequencing after having reached the stationary phase (Fig. 1). All strains grew rather similarly in the WSH; growth was resumed after a lag time of ~10 h (Fig. 1). Expectedly, as *S. cerevisiae* is auxotroph for ergosterol when grown anaerobically (35), only 2–3 doublings were observed before the cells entered the stationary phase. Considering the differences in growth of the different strains in aerobic conditions, the similar growth patterns and fermentation profiles in anaerobic conditions were not expected. Notably, no statistical differences were seen within the physiological parameters measured for the two LBCM strains (Table 2). This simplified the comparison of the DEGs among these two strains as strain physiology at the sampling time *per se* was not expected to reflect their transcriptomes.

**TABLE 2:** DCW, maximal growth rate (µmax), and final metabolite concentration of the strains^*a*^

Strain	DCW (g L^−1^)	µmax (h^−1^)	Glucose (g L^−1^)	Xylose (g L^−1^)	Xylitol (g L^−1^)	Acetate (g L^−1^)	Ethanol (g L^−1^)
CEN.PK113-7D	2.7 ± 0.5	0.16 ± 0.1	0.7 ± 0.1	19.6 ± 0.3	0.6 ± 0.0	7.0 ± 0.0	32.8 ± 0.9
KE6-12	3.7 ± 0.3	0.15 ± 0.0	0.5 ± 0.0	15.9 ± 0.6	0.8 ± 0.2	5.6 ± 0.3	33.6 ± 0.9
Ethanol Red	3.2 ± 0.1	0.17 ± 0.0	0.6 ± 0.0	19.4 ± 0.1	0.3 ± 0.2	5.5 ± 0.4	32.2 ± 0.8
LBCM31	3.7 ± 0.2	0.20 ± 0.0	0.0 ± 0.0	19.4 ± 0.3	0.6 ± 0.1	5.0 ± 0.6	29.2 ± 1.5
LBCM109	3.6 ± 0.4	0.20 ± 0.0	0.0 ± 0.0	19.4 ± 0.0	0.6 ± 0.1	5.8 ± 0.3	31.6 ± 0.6

^*a*^ Data presented are the average of four biological replicates ± standard deviation. Also arabinose, HMF, and furfural were measured. Arabinose was not consumed by any strain, and HMF and furfural levels were below the detection level for all strains.

The biomass accumulation and µmax of the strains spanned from 2.7 ± 0.5 to 3.7 ± 0.3 g DCW L^−1^ and from 0.15 to 0.20 h^−1^, respectively (Table 2). Notably, the LBCM strains had a higher µmax compared with all other strains. At the time of harvest, all strains had consumed all or almost all the glucose and accumulated similar amounts of ethanol, ranging from 29.2 ± 1.5 to 33.6 ± 0.9 g L^−1^. The 70% WSH media used for this study contained 5.9 g L^−1^ acetic acid, and this concentration was retained or even slightly diminished in most cultures (Table 2). Only trace amounts of xylose were converted to xylitol with the exception of the xylose-utilizing KE6-12 strain that had consumed ~4 g L^−1^ of xylose at the time of sampling (Table 2). Xylitol is produced from xylose by the native aldose reductase Gre3 (36) or in KE6-12 by the heterologously expressed xylose reductase, Xyl1. Both enzymes are NADPH dependent, and as NADPH is produced predominantly through the aerobic pentose phosphate pathway, this is likely to explain the modest xylitol production and xylose consumption of the strains.

### Unsupervised and DGE analyses showed significant differences in gene expression between the strains

**Fig 2:**
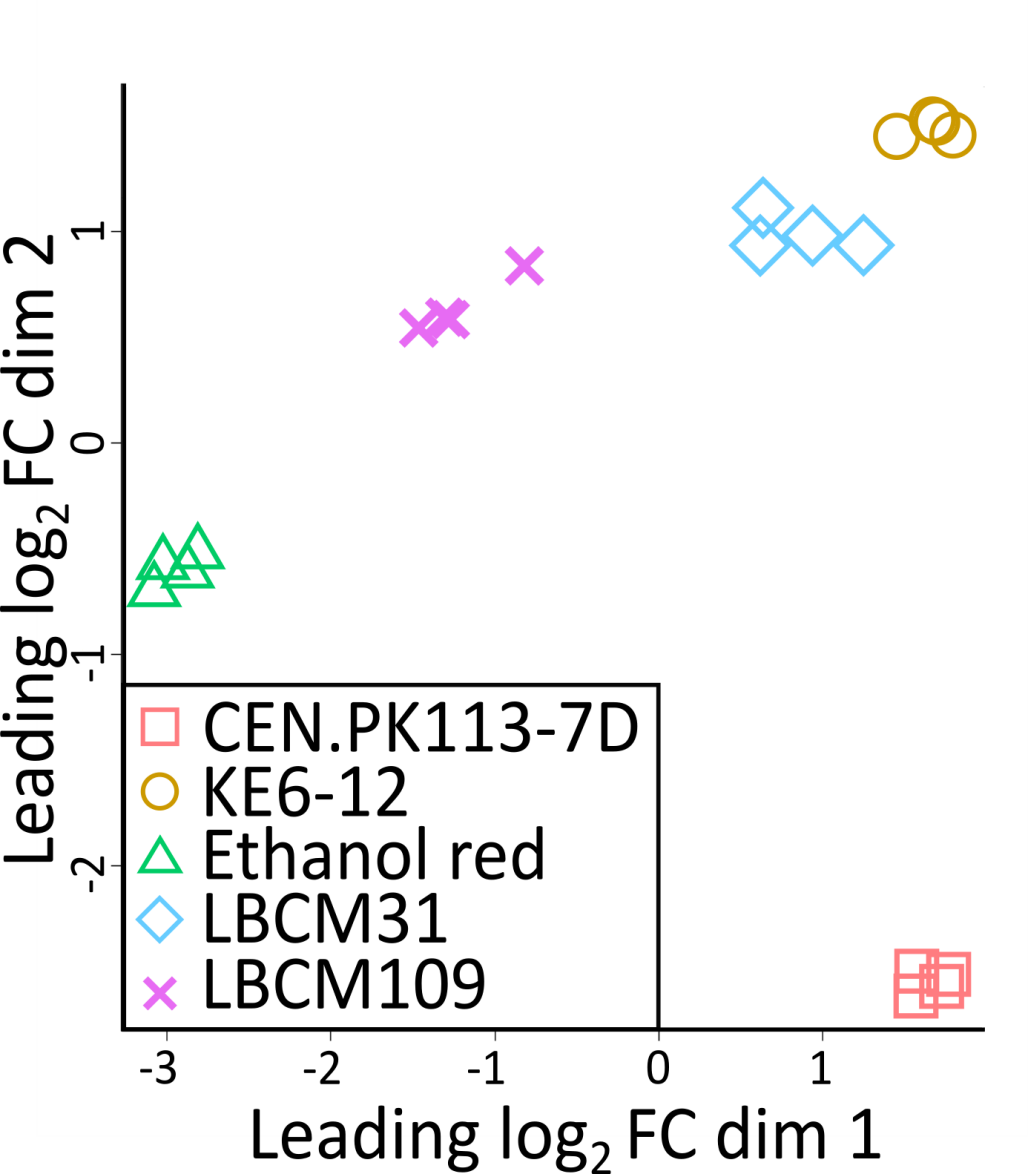
Unsupervised multi-dimensional scaling plot of all RNA sequencing samples of CEN.PK113-7D (red squares), KE6-12 (yellow circles), Ethanol Red (green triangles), LBCM31 (blue diamonds), and LBCM109 (purple crosses). X and Y axes represent the first (dim 1) and second (dim 2) leading fold change that best separates the samples and explains the largest proportion of variation in the data.

Unsupervised multi-dimensional scaling analysis revealed that the four replicate samples grouped together and also apart from other sample replicates (Fig. 2). This indicated a good reproducibility of the results, as well as a substantial difference in transcriptomic signature between the different samples. This presumably reflects the different genetic backgrounds of the strains. The levels of non-aligned sequences were similar for all strains; less than 10% of the sequences did not map to any loci.

**Fig 3:**
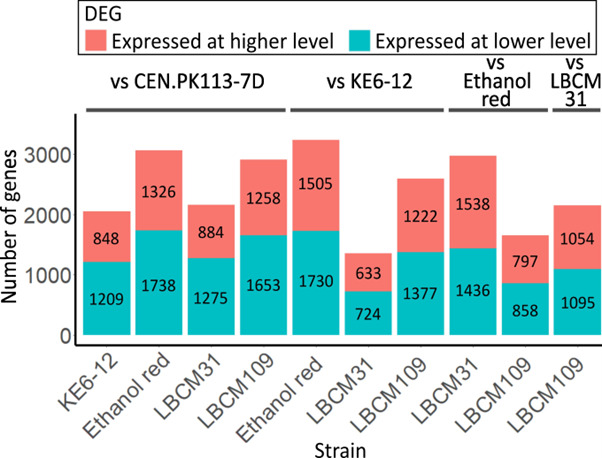
Counts of the significant DEGs between the strains analyzed. The number of genes that were expressed at a significantly higher (red bars) or lower (green bars) level for the strain reported at the bottom of the bars compared with the one specified at the top; CEN.PK113-7D, KE6-12, Ethanol Red, and LBCM31. Significance was defined as adjusted *P* value < 0.01 and fold change ≥ 2. Data presented are based on the average of four biological replicates.

A large number of significantly (adjusted *P*-value < 0.01; fold change ≥2) DEGs were identified—from 1,357 in the comparison between LBCM31 and KE6-12 to 3,235 in the comparison between KE6-12 and Ethanol Red (Fig. 3). Considering the different genetic background of the strains analyzed, a high number of DEGs was expected. It should be noted that the RNA sequences of all strains were aligned to the same reference genome. This could potentially partially attribute to larger genome differences that could impact alignment of reads. Major differences in gene expression among different *S. cerevisiae* strains have been reported earlier (37, 38). van Dijk et al. (16) identified 1,162 DEGs between *S. cerevisiae* cells of the same strain when cells adapted to lignocellulosic hydrolysate were compared with non-adapted cells. This study showed that a large amount of genes can be involved in the adaptive response. On the other hand, when the transcriptional response of *S. cerevisiae* T2 in the presence of individual inhibitors was compared with the transcription of cells in the presence of hardwood spent sulfite liquor, merely 400 genes showed significant expression changes (15). This emphasizes that the number of DEGs can be strain and condition dependent.

### Pairwise comparisons of strains revealed large overlaps in the most significant DEGs of the LBCM strains compared with the other strains

There was a large overlap between the genes that were expressed at the highest or lowest level in the LBCM strains, when compared with the other analyzed strains (supplementary materials: Fig. S1; Table S1). Considering all comparisons including the 10 most significant DEGs, a total of 29/60 genes were common for both strains. All common genes expressed at the lowest level (*GPP2*, *HOM3*, *MAL12*, *PRM7*, and *YAR028W*) and two of the common genes expressed at the highest level (*GRE1* and *GTT1*) in the LBCM strains compared with the CEN.PK113-7D strain have previously been identified to play a part in hydrolysate-related stress responses (1). *HOM3* and *PRM7* are both regulated by Gcn4, a transcriptional activator of amino acid biosynthetic genes. Genes involved in biosynthesis of amino acids are well known to be involved in the tolerance to lignocellulosic inhibitors (1, 15). *GPP1* and *GPP2* were expressed at a 1.6- or 3.8-fold lower level in the LBCM strains compared with the CEN.PK strain (supplementary materials: Fig. S1; Table S1). *GPP1* and *GPP2* encode glycerol phosphatases which are induced in response to osmotic stress (39), and overexpression of *GPP2* was shown to increase tolerance to inhibitors in hydrolysates (2).

*GRE1*, which was among the highest expressed genes in the LBCM strains compared with CEN.PK113-7D, encodes a stress-induced hydrophilin. The expression of *GRE1* was previously found to be downregulated in an industrial strain adapted to growth in lignocellulosic hydrolysates (16). Still, in other studies (and strains), *GRE1* was found significantly upregulated in the presence of a mix of inhibitors common in hydrolysates (40, 41). The function of Gre1 has not been elucidated, but its paralogue, Sip18, was reported to be a cytoplasmic phospholipid-binding protein. The expression of *SIP18* was shown to be induced by osmotic stress (42). In our study, *SIP18* was highly expressed in both LBCM strains when compared with the CEN.PK strain (supplementary materials: Table S1). The high expression of *GRE1* and *SIP18* may thus be a means for counteracting osmotic stress in the LBCM strains, whereas the CEN.PK strain may be more prone to regulate its glycerol synthesis for achieving osmotolerance. In anaerobic conditions, glycerol production is essential to reoxidize NADH and glycerol is produced to counteract osmotic stress. Growth in lignocellulosic hydrolysates as well as ethanol accumulation is reported to cause osmotic stress in yeast (43).

The LBCM strains were isolated from a cachaça distillery where they have adapted to high ethanol concentrations and osmotic stress. A recent study comparing the genetic variation among 11 strains of the LBCM collection shared single-nucleotide variants of many genes encoding proteins involved in the tolerance to fermentative stresses and ethanol (44). This adaptation to ethanol may also explain the high expression levels of *ZNF1* and *AQY3*, which were among the most highly expressed genes in the LBCM strains compared with KE6-12 (>3.5-fold increase, Fig. S1; Table S1). *ZNF1* encodes a zinc cluster transcription factor required for adaptation to pH, osmotic, and ethanol stress (45). The overexpression of *ZNF1* has been shown to increase acetic acid tolerance and improve ethanol productivity (46). Aqy3 is an aquaporin, similar to Fps1 that plays a critical role in osmoregulation by controlling the accumulation of the osmolyte glycerol but also small molecules such as acetate (47). Much less is known about Aqy3 compared with Fps1, but a recent study revealed that *AQY3* was mutated in a strain resistant to low pH, elevated acetic acid concentrations, and high temperature (48). This indicates that Aqy3 indeed can play a role in resistance to stressors that are present in lignocellulosic hydrolysates. Changing the cellular uptake or export of inhibiting compounds can function as a complement to inhibitor detoxification inside the cells.

*S. cerevisiae* is able to detoxify formic acid to CO_2_ by formate dehydrogenases such as Fdh1, Fdh2, and YPL276W. *FDH1* was among the genes whose relative expression in the LBCM strains compared with both KE6-12 or Ethanol Red was lowest (Fig. S1; Table S1). Similarly, the expression of *FDH2* and *YPL276W* was expressed at a lower level in both LBCM strains when compared with KE6-12. We suggest that the higher expression of formate dehydrogenase-encoding genes may be a result of the strain improvement KE6-12 or Ethanol Red have gone through. Adaptive laboratory evolution to improve formate tolerance in *S. cerevisiae* CEN.PK 113-5D led to a ~ 3,000-fold higher expression of the formate dehydrogenase-encoding genes *FDH1*, *YPL276W*, and *FDH2* (49). Overexpression of *FDH1* has been demonstrated to increase tolerance to formic acid and acetic acid, through decomposition of formic acid and generation of additional ATP, respectively (50).

### The LBCM strains showed high expression of glutathione-related genes

**Fig 4:**
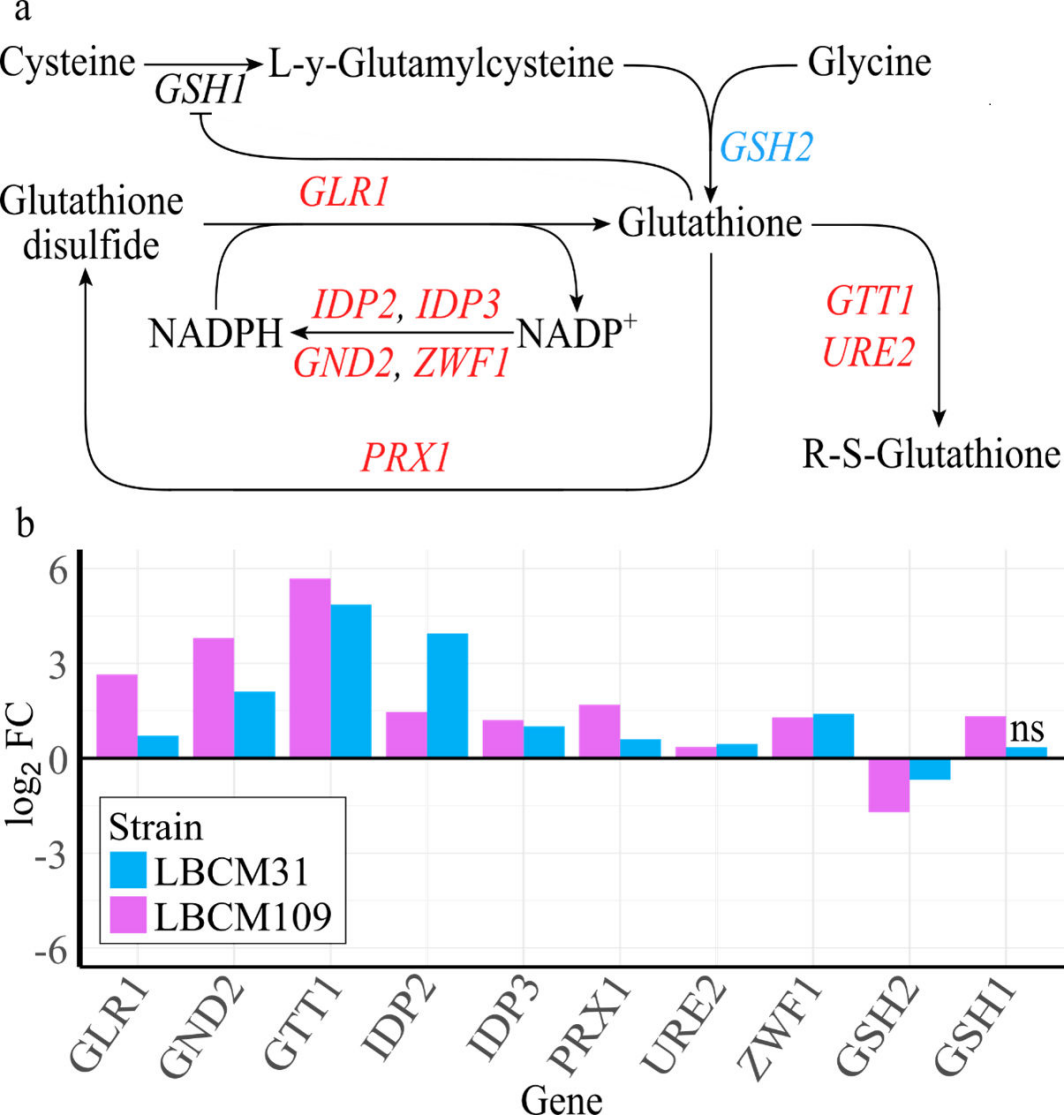
Expression of genes related to glutathione metabolism and NADPH regeneration. (a) Schematic map depicting the metabolic pathway of glutathione according to the KEGG pathway representation. Elements in pink and blue represent genes that are expressed at a significantly (adjusted *P* value <  0.01) higher or lower level in both LBCM strains compared with CEN.PK113-7D. (b) Differential expression of genes related to glutathione metabolism in LBCM31 (blue bars) and LBCM109 (purple bars) compared with CEN.PK113-7D. The relative expression level of each gene is visualized as log2 of the fold change (log2 FC). The letters “ns” above the last bar represent the statistically non-significant (adjusted *P* value >  0.01) differential gene expression for that comparison.

Several genes involved in glutathione metabolism (and NADPH regeneration) were expressed at a significantly higher level in both LBCM strains compared with CEN.PK, namely, *GTT1*, *URE2*, *GLR1*, *IDP3*, *IDP2*, *GND2*, *ZWF1*, and *PRX1* (Fig. 4). On the contrary, *GSH2* was expressed at a significantly lower level (Fig. 4). *GSH1*, encoding a glutamylcysteine synthetase that catalyzes the first and rate-limiting step in the glutathione biosynthetic pathway, was highly expressed in LBCM109 but not differentially expressed in LBCM31 compared with CEN.PK (supplementary materials: Table S1). *GSH2* encodes an ATP-dependent glutathione synthase and while *GSH1* overexpression was shown to increase glutathione content in cells, the deletion of *GSH2* was shown to have no impact on the resistance to oxidative stress (51). Increasing the glutathione content in yeast was shown to increase tolerance toward lignocellulose inhibitors (52). Many genes involved in glutathione metabolism have been reported to be upregulated during formic acid treatment (53). Oxidative stress due to accumulation of reactive oxygen species generated during aerobic growth in the presence of lignocellulosic hydrolysates is well documented. Moreover, the presence of furfural has been shown to lead to oxidative stress (54) and yeast has been suggested to suffer from oxidative stress also during anaerobic fermentation (55). Yeast cells lacking glutathione have been shown to be sensitive to oxidative stress (56).

Our data allows us to hypothesize that the LBCM strains have evolved to recycle glutathione rather than to produce more glutathione. *GTT1* encoding a glutathione transferase was the most highly expressed gene when comparing the LBCM strains to CEN.PK. Glutathione transferases function to detoxify the cells against for instance xenobiotics, environmental pollutants, or harmful small molecules (57); thus, it seems plausible that Gtt1 could detoxify inhibitors found in lignocellulosic hydrolysates. *GTT1* was previously reported to be upregulated in evolved strains exposed to inhibitors (14). Similarly, high expression of *PRX1* encoding a peroxiredoxin that upon oxidative stress transfers oxidative equivalents to glutathione that is oxidized to glutathione disulfide (58) could provide a means for the LBCM strains to ease their oxidative stress. Recently, oxidized glutathione was demonstrated to play a key role in the response of yeast to formic acid stress (53). Overexpression of *GLR1* encoding a glutathione oxidoreductase was on its own shown to not impact glutathione contents of cells, possibly due to limitation of NADPH (52). Thus, the high expression of not only *GLR1* but also many genes encoding enzymes responsible for NADPH regeneration (e.g., *IDP2*, *IDP3*, GND2, and *ZWF1*) may lead to increased glutathione-dependent antioxidant activity in the LBCM strains.

### Many GO terms previously associated with importance in hydrolysate tolerance were among the DEGs of the LBCM strains

**Fig 5:**
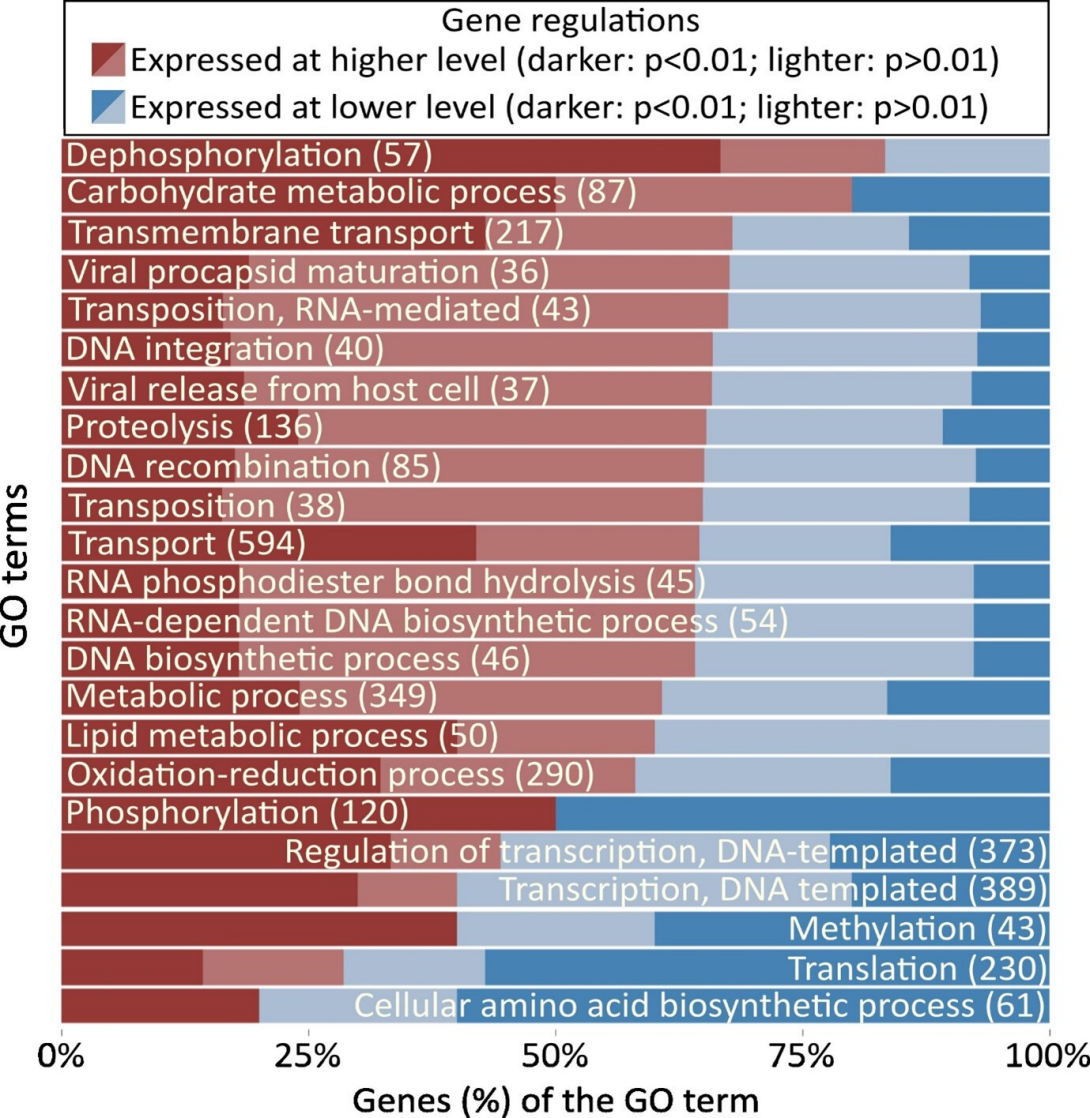
GO term analysis of genes differently expressed in LBCM109 when compared with LBCM31. Percentages of expressed genes at a significantly (adjusted *P* value < 0.01) higher or lower level are marked in dark-red or dark-blue, respectively, whereas genes differently expressed, although not at a significant level (adjusted *P* value > 0.01), are marked in light-red or light-blue, respectively. The name of each GO term is inside the left or the right side of its relative bar, depending on whether the majority of the genes for that GO term are expressed at a higher (left side) or lower (right side) level. Each GO term name is followed by the total number of genes of that GO term. Data obtained from four biological replicates.

While the LBCM strains were both isolated from cachaça distilleries and showed many similarities in terms of transcriptional response to lignocellulosic hydrolysates compared with the other strains analyzed, they still had ~2,000 DEGs (Fig. 3). Therefore, we did a comparative GO enrichment analysis on the DEGs of the two LBCM strains. A total of 23 GO terms were identified for the DGEs, and 13 of these referred to DNA or RNA processes (Fig. 5; supplementary materials: Table S2). All GO categories enriched contained DEGs expressed at both higher and lower levels in LBCM109 compared with LBCM31 (Fig. 5). In line with this, studies where the EUROSCARF mutants were profiled for resistance to inhibitors often demonstrated a great antagonism in the genes leading to increased tolerance towards inhibitors (1). Notably, a few GO terms that were enriched during stress caused by lignocellulosic inhibitors in the study of Vanacloig-Pedros et al. (59) were similar to the GO terms of the DGEs we noted when comparing LBCM31 to LBCM109, namely, “RNA processing and translation,” “amino acid biosynthesis and mitochondrial stress,” and “transcription regulation.” Transcription, translation, and amino acid synthesis-related GO terms were enriched for genes that were predominantly expressed at a lower level in LBCM109 compared with LBCM31 (Fig. 5). In line with this, a transcriptomic study with a *S. cerevisiae* strain adapted to spent sulfite liquor concluded that acetic acid and HMF stress affected genes associated with biosynthesis of amino acids (15). Modification of amino acid synthesis genes or overexpression of transcription factors has in many studies proven to be a successful strategy for improving tolerance toward inhibitors (1).

**Fig 6:**
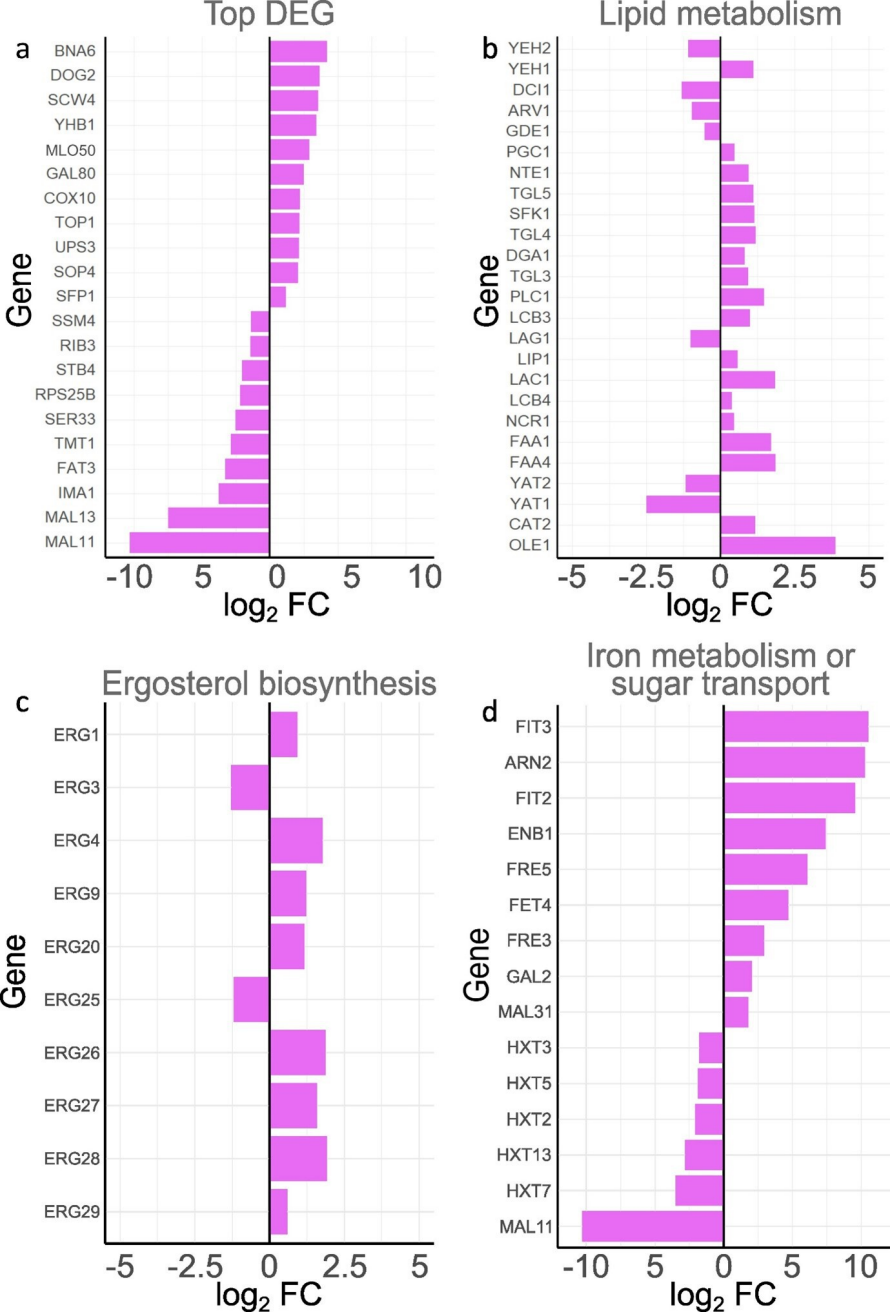
Log_2_ FC of the (a) 10 genes expressed at highest or lowest level; (b) most significantly differentially expressed genes of the “lipid metabolism” GO term; (c) most significantly differentially expressed genes involved in ergosterol biosynthesis; (d) most significantly differentially expressed genes involved in iron metabolism or sugar transport in LBCM109 when compared with LBCM31. Significance was defined as adjusted *P* value < 0.01. Data are obtained from four biological replicates. All data on the DEGs are found in Tables S3-S4.

Among the enriched GO terms that predominantly contained genes expressed at a higher level in LBCM109 compared with LBCM31, we noted many terms describing metabolic processes, such as “dephosphorylation,” “carbohydrate metabolic process,” “transmembrane transport,” “lipid metabolic process,” and oxidation-reduction process” or DNA modulation, such as “DNA integration,” “transposition,” and “DNA recombination.” Similarly, comparative transcriptomics of two strains evolved in lignocellulosic hydrolysates revealed 52 DEGs in medium with multiple inhibitors, >50% of which clustered in the GO term “metabolic process” that contains genes related to fatty acid metabolism, general cellular metabolism, and oxidative stress response (14). A large set of genes related to oxidative stress response was induced by propagation in lignocellulosic hydrolysates, further demonstrating their importance in hydrolysate tolerance (16). In summary, many of the GO terms enriched for genes that were differently expressed between the two LBCM strains were previously highlighted in studies on tolerance to hydrolysates or inhibitors therein, indicating that stress mechanisms typically identified during aerobic conditions may also be important for anaerobic processes. Almost all of the 10 most differently expressed genes of the LBCM strains (Fig. 6A) have been previously reported to be important for tolerance to hydrolysate or inhibitors therein [*BNA6* (60), *YHB1* (61), *COX10* (62–65), *SCW4* (2, 65), *TOP1* (2, 66), *UPS3* (2, 65), *SOP4* (2, 65, 66), *SFP1* (64), *SSM4* (66), *STB4* (2, 65), *SER33* (65), *TMT1* (65), *FAT3* (65), *IMA1* (65), *MAL13* (2, 65), and *MAL11* (67)] or osmotic and oxidative stress tolerance [*DOG2* (68)]. Still, their mechanistic role in this context is often still to be elucidated. It should however be noted that the genetic background of a strain may strongly influence how a specific gene influences strain physiology. Furthermore, the results from screens of the EUROSCARF deletion collection for tolerance to individual or mixed inhibitors (2, 62, 64, 65) have been shown to be highly context dependent (1). Therefore, we here highlight similarities among the LBCM strains that were previously identified across studies.

### Cell wall-related genes and genes involved in lipid and membrane biosynthetic genes were upregulated in LBCM109 compared with LBCM31

Genes falling under the GO term “cell wall function” have previously been reported to be important for acetic acid tolerance (65). In our study, 3 of the 10 most upregulated genes in the carbohydrate metabolic process GO term that was enriched for LBCM109, *SCW4*, *GAS2*, and *GAS5* encode proteins important for the cell wall (Table S4). *SCW4* encodes a cell wall protein, whereas *GAS2* and *GAS5* encode 1,3-beta-glucanosyltransferases. *GAS2* was reported to be expressed exclusively during sporulation while *GAS5* is expressed during vegetative growth (69). The overexpression of *GAS2* was demonstrated to decrease growth in a medium supplemented with acetate (65) or lignocellulosic inhibitors (2). *GAS5* deletion on the other hand was shown to increase acetate tolerance of a laboratory strain (65). The single deletion of *SCW4* or *GAS5* has been shown to alter the cell wall (70). The cell wall, together with the plasma membrane, is the first barrier of *S. cerevisiae*, and its structure is very important for the resistance to lignocellulosic hydrolysates [reviewed in reference (71)].

Out of the 50 genes comprised in the “lipid metabolism” GO term, 28 were differentially expressed in LBCM109 and LBCM31 (Fig. 6B). Those genes encode proteins regulating the metabolism and transport of a variety of lipids, including fatty acids (*OLE1*, *CAT2*, *YAT1*, and *YAT2*), sphingolipids (*FAA4*, *FAA1*, *NCR1*, *LCB4*, *LAC1*, *LIP1*, *LAG1*, and *LCB3*), acylglycerols (*PLC1*, *TGL3*, and *DGA1*), phospholipids (*TGL4*, *SFK1*, *TGL5*, *NTE1*, *PGC1*, *GDE1*, *ARV1*, and *DCI1*), and sterols (*YEH1*, *YEH2*) (Fig. 6B; supplementary material: Tables S3 and S4). Notably, many of the genes that were expressed at a higher level in LBCM109 when compared with LBCM31 encode proteins involved in synthesis and accumulation of sphingolipids or phospholipids. Sphingolipids as well as phospholipids are crucial components of the plasma membrane as well as other cellular membranes [reviewed by Hannun and Obeid (72)]. Previous studies revealed a link between a high level of complex sphingolipids and the natural tolerance of *Zygosaccharomyces bailii* to acetic acid (73–75). Furthermore, lipid remodeling of *S. cerevisiae* upon exposure to weak acids has been found to result in the increase of very-long-chain fatty acids, which are the precursors of sphingolipids (76). Guo et al. also uncovered a change in phospholipid composition following acid stress and found that overexpression of *OLE1* led to an increased unsaturation index of fatty acids in the plasma membrane and a higher tolerance to acetic, formic, and levulinic acids. *OLE1* that encodes an essential ∆-9 fatty acid desaturase required for the production of monounsaturated fatty acids was the most highly expressed gene for the “lipid metabolism” GO term in LBCM109 when compared with LBCM31. Similarly, *FAA1* and *FAA4* that are paralogs encoding long-chain fatty acyl-CoA synthases were highly expressed in LBCM109 (Fig. 6B). While the deletion of *FAA1* was shown to increase tolerance to acetic acid (65), the Δ*faa1* strain was more sensitive to formic acid (10). Deletion of *FAA1* and *FAA4* has been demonstrated to be an effective way to increase the level of free fatty acids in yeast (77), and sensitivity to oxidative stress in both aerobic and anaerobic conditions has been shown to be dependent on the membrane lipid composition (78). Also, genes involved in ergosterol biosynthesis, *ERG27*, *ERG4*, *ERG28*, *ERG26*, *ERG9*, *ERG20*, *ERG1*, and *ERG29*, were expressed at a higher level in LBCM109 compared with LBCM31 (Fig. 6C; supplementary material: Table S3). The ergosterol content of the cell membrane of *S. cerevisiae* has been reported to change under stress caused by organic acids (76). The deletion of individual ergosterol synthesis genes has been reported to alter tolerance to acetic and formic acids (1). Studying the lipid composition of the two LBCM strains could shed light on the role of lipid metabolism in the tolerance to lignocellulosic hydrolysates.

### Various transporter-encoding genes were differently expressed in the two LBCM strains

Several genes encoding transporters have been reported to be involved in yeast tolerance to inhibitors or lignocellulosic hydrolysates (1). The importance of transporters was also highlighted in our study. The LBCM strains showed great differences in expression of genes of the GO terms “transport” (GO:0006810) and “transmembrane transport” (GO:0055085) (Fig. 6D; supplementary materials: Table S4). A total of 347 out of the 594 genes belonging to the GO term “transport” and 124 out of the 217 genes falling under the GO term “transmembrane transport” were significantly differentially expressed in LBCM31 and LBCM109 (supplementary materials: Table S4).

Five of the 20 most highly expressed transport genes in LBCM31 compared with LBCM109 were members of the hexose transporter family: *HXT2*, *HXT3*, *HXT5*, *HXT7*, and *HXT13* (Fig. 6D, supplementary materials: Table S4). Moreover, the maltose and trehalose transporter-encoding gene *MAL11* was the most highly expressed transporter-encoding gene in LBCM31 when compared with LBCM109. Overexpression of *MAL11* was shown to improve xylose uptake in *S. cerevisiae* (79). *GAL2* encoding another hexose transporter, a galactose permease important for uptake of xylose (80), as well as another maltose transporter-encoding gene, *MAL31*, and the hexose transporter genes *HXT6* and *HXT12* were on the other hand expressed at a higher level in LBCM109 (Table S3). Overexpression of hexose transporters has been proven to lead to higher glucose (81) or xylose [reviewed in reference (82)] uptake and improved cell growth in *S. cerevisiae*. A higher rate of glucose intake may help the cell in producing more energy as well as cofactors for inhibitor tolerance and detoxification (83). Furthermore, a faster glucose depletion may push the cell to consume sooner alternative carbon sources, such as acetic acid, hence contributing to a quicker detoxification of the media. Indeed, LBCM31 cultures displayed a slightly lower amount of acetic acid at the end of the cultivation compared with LBCM109 (Table 2). Thus, the two LBCM strains may have evolved different strategies for efficient sugar uptake.

Seven of the 10 most highly expressed genes belonging to the GO term “transport” in LBCM109 encode proteins involved in iron transport and homeostasis; Fit2, Fit3, Fre3, Fre5, Fet4, Arn2, and Enb1 (Fig. 6D). Also *CCC1* and *MRS3*, encoding a vacuolar and a mitochondrial iron transporter, respectively, were expressed at a significantly higher level in LBCM109 compared with LBCM31 (Table S3). Iron is found in various biomolecules and is essential for all cells, whereas excessive iron levels are toxic, both in aerobic and anaerobic conditions (84). Many iron metabolism-related genes have been previously reported to be involved in tolerance toward lignocellulosic hydrolysate inhibitors (1). *FIT2* and *FIT3* encode mannoproteins involved in the retention of siderophore-iron in the cell wall (85). Several iron transport-related genes, including *FIT2* and *FIT3*, were upregulated in two *S*. *cerevisiae* strains upon exposure to furfural (86). The authors hypothesized that the high expression of siderophore iron transmembrane transporter-encoding genes may be a means for adaptation to a higher, inhibitor-induced, demand of iron (86). They also mention iron leakage due to membrane damage as a possible explanation for increased need of iron transporters, which could also explain why the LBCM109 strain had high expression of ergosterol genes (Fig. 6C).

### Conclusions

The LBCM strains had a higher µmax compared with the other strains when grown anaerobically in the presence of lignocellulosic hydrolysate. Our strain comparison demonstrates that naturally tolerant strains can be good alternatives to strains adapted to a specific substrate. When compared with the industrial strains or CEN.PK113-7D, the LBCM strains also shared many transcriptomic responses. Numerous genes showing differential expression among the strains have previously been recognized as crucial for tolerance to lignocellulosic hydrolysates or their inhibitors. This underscores that stress-related mechanisms identified under aerobic conditions also play a pivotal role in anaerobic processes. Collectively, the work sheds light on strain-specific mechanisms regulating lignocellulosic hydrolysate tolerance and improves our comprehension of stress resistance in yeast. This can be applied to improve the stress tolerance of *S. cerevisiae* for biorefinery applications.

## Data Availability

The data sets supporting the conclusions of this article are included within the article and its additional files. The corresponding author is willing to provide the raw data related to this article upon reasonable request.

## SUPPLEMENTAL MATERIAL

Figure S1 aem.02330-23-s0001.docx Expression level of the common genes, among the 10 most significantly differentially expressed in LBCM31 and LBCM109, when compared to the other strains.

Table S1 aem.02330-23-s0002.xlsx Gene expression in LBCM strains compared to the other strains.

Table S2 aem.02330-23-s0003.xlsx Most enriched GO terms in LBCM strains.

Table S3 aem.02330-23-s0004.xlsx Differential gene expression analysis between LBCM109 and LBCM31.

Table S4 aem.02330-23-s0005.xlsx Differential gene expression analysis of the GO terms highlighted in Fig. 5 between LBCM109 and LBCM31.

ASM does not own the copyrights to Supplemental Material that may be linked to, or accessed through, an article. The authors have granted ASM a non-exclusive, world-wide license to publish the Supplemental Material files. Please contact the corresponding author directly for reuse.
