# Supplementary material for: Saccharomyces cerevisiae strains performing similarly during fermentation of lignocellulosic hydrolysates show pronounced differences in transcriptional stress responses
Source: Appl Environ Microbiol. 2024 Apr 8;90(5):e02330-23. doi: 10.1128/aem.02330-23 (PMC11107148; doi:10.1128/aem.02330-23)
Supplement: Figure S1 — Expression level of the common genes, among the 10 most significantly differentially expressed in LBCM31 and LBCM109, when compared to the other strains. [file aem.02330-23-s0001.docx]

**Supplementary figure S1.**

S*accharomyces cerevisiae* strains performing similarly during fermentation of lignocellulosic hydrolysates show great differences in transcriptional stress responses

Elena Cámara, Maurizio Mormino, Verena Siewers, Yvonne Nygård


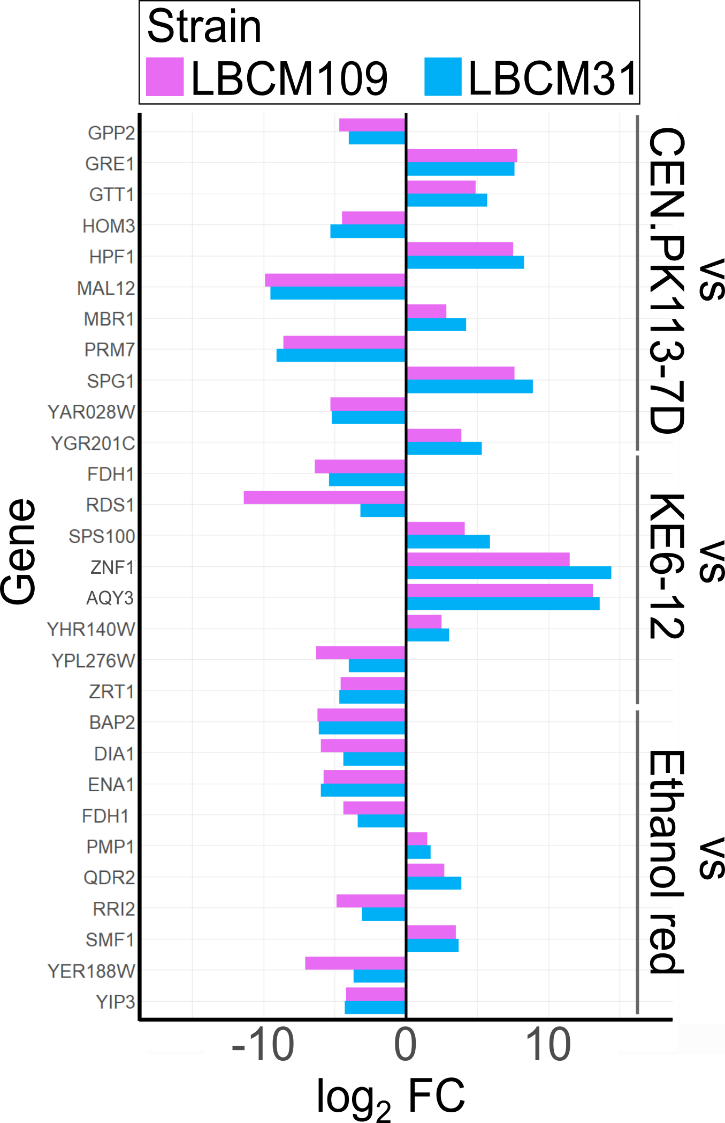


Figure S1: Expression level of the common genes, among the 10 most significantly differentially expressed in LBCM31 (blue bars) and LBCM109 (purple bars), when compared to the other strains (CEN.PK113-7D, KE6-12 and Ethanol). The expression level of each gene is visualized as log_2_ of the fold of change (log_2_ FC). Significance was defined as adjusted p value < 0.01 and fold of change ≥ 2. Data obtained from four biological replicates. All data on the DEGs are found in the supplementary material, Table S1.
